# 

**Published:** 1881-10

**Authors:** 


### THE BISTOURY:

Thad S. Up de Graff, M. D., Editor & Proprietor.

### *CONTENTS FOR OCTOBER, 1881.*

**CONTRIBUTIONS.** PAGE.

Cheek—Does It Pay? ....... BEN. H. PRATT ....... 53
Doctors, Ancient and Modern ....... AUSBURN TOWNER ....... 55
Hereditary ....... J. B. CHANDLER ....... 57
State Education ....... THEOPHRASTUS SCHOPENHAVER ....... 59
Bacteria ....... S. O. GLEASON ....... 60

**MEDICAL ITEMS AND NEWS.**

Chilblains—Diarrhœa—Cholera Mixture—Coughs and Night Sweats of Phthisis—Cholera Infantum—Cystitis—Inflamation of the Mammae—Acute Catarrh—Chronic Malarial Poisoning—Summer Dysentery—Facial Erysipelas—Cholera Mixture—Quebracho—Eclampsia—Itch—Erysipelas—Freckles—Bilious Colic—Croupous Pneumonia—Eclampsia—Asthma—Sore Nipples—Hysteria and Epilepsy—Growth of Hair—Asthmatic Paroxism—Chronic Rheumatism—Acute Bronchitis—Summer Diarrhœa—Rhus Aromatica—Prolapsus Ani—Constipation—Sub-Acute Pleurisy—Gout—Tetanus—Asthma—Diphtheria—Restoring the Heart's Action—Billiary Calculb—Diphtheria—Affections of the Skin—Burns ....... 62 68

**OUR CORRESPONDENCE** Six Letters with answers ....... 69

**HOUSEHOLD HINTS AND RECIPES.**

To Remove Old Ink-Sbots—Fire-Proof Cement—Charcoal Tooth Powder—Oxidizing Silver—Ladies' Shoe Dressing—Rapid Germination of Seeds—To Preserve Starch Paste—Varnish for Preventing Rust—Dangers of Mouldy Bread—To Render Leather Water-Proof—Liquid Starch Gloss—Liquid Glue—A New Dye for Feathers—Coffee and Egg for Sick Persons—The Treatment of Toothache ....... 70 71

**EDITORIAL DEPARTMENT.**

A Nation Mourns—The Nineteenth of the Month—The North American Review—Mis-Named—Dr. Bliss and Dr. Hammond—Cooling a Watermelon—Probing for Bullets—That "Induction Balance"—The Contributions—A Beautiful Simile—The Bishop and the Squirrel—A Silver Medal—Emphatically Settled—The American Society of Microscopists—Second Sight—Guiteau—Our Sewerage—The Attending Surgeons—Not an Evil—Whistling—The State Fair—Pay Up—Dr. Geo. E. Blackham—Gambling—Worthy of Record ....... 72 78

